# Homogeneity of the 16S rDNA sequence among geographically disparate isolates of *Taylorella equigenitalis*

**DOI:** 10.1186/1746-6148-2-1

**Published:** 2006-01-06

**Authors:** M Matsuda, A Tazumi, S Kagawa, T Sekizuka, O Murayama, JE Moore, BC Millar

**Affiliations:** 1 Department of Bacteriology, Northern Ireland Public Health Laboratory, Belfast City Hospital, Belfast BT9 7AD, Northern Ireland, UK.; 2 School of Biomedical Sciences, University of Ulster, Cromore Road, Coleraine, Co. Londonderry, BT52 1SA, Northern Ireland, UK.; 3 Laboratory of Molecular Biology, School of Environmental Health Sciences, Azabu University, Fuchinobe 1-17-71, Sagamihara 229-8501, Japan.

## Abstract

**Background:**

At present, six accessible sequences of 16S rDNA from *Taylorella equigenitalis *(*T. equigenitalis*) are available, whose sequence differences occur at a few nucleotide positions. Thus it is important to determine these sequences from additional strains in other countries, if possible, in order to clarify any anomalies regarding 16S rDNA sequence heterogeneity. Here, we clone and sequence the approximate full-length 16S rDNA from additional strains of *T. equigenitalis *isolated in Japan, Australia and France and compare these sequences to the existing published sequences.

**Results:**

Clarification of any anomalies regarding 16S rDNA sequence heterogeneity of *T. equigenitalis *was carried out. When cloning, sequencing and comparison of the approximate full-length 16S rDNA from 17 strains of *T. equigenitalis *isolated in Japan, Australia and France, nucleotide sequence differences were demonstrated at the six loci in the 1,469 nucleotide sequence. Moreover, 12 polymorphic sites occurred among 23 sequences of the 16S rDNA, including the six reference sequences.

**Conclusion:**

High sequence similarity (99.5% or more) was observed throughout, except from nucleotide positions 138 to 501 where substitutions and deletions were noted.

## Background

Contagious equine metritis (CEM), which was first reported in thoroughbred mares during the 1977 breeding season in the UK [1], is an important bacterial genital infectious disease of horses caused by *Taylorella equigenitalis*. CEM generally leads to a loss of fertility in mares and the disruption of breeding programs. Since the first report of CEM, this disease and its causative agent have been detected in many countries and in various breeds of horses [2,3]. Diagnosis of CEM has been performed by the isolation of *T. equigenitalis*, a nonmotile gram-negative coccobacillus, cultured by means of conventional selective bacteriological techniques.

In relation to the phylogenetic positioning of *T. equigenitalis*, Bleumink-Pluym *et al*. were the first to determine 16S rDNA sequences from three strains of *T. equigenitalis *(NCTC11184^T^, N480/82 and N610/88; GenBank Accession No. X68645) to be identical and demonstrated this organism to belong to the β subclass of the class *Proteobacteria *[4]. When Jang *et al*. analyzed the phylogenetic position of *T. asinigenitalis*, which is a new second species within the genus *Taylorella*, and a gram-negative, non-motile coccobacilli but phenotypically indistinguishable from *T. equigenitalis*, which was isolated from donkey jacks (*Equus asinus*) in the USA, in 1997 and 1998 [5], they described that *T. equigenitalis *appears to be homogeneous with respect to the 16S rDNA sequence, since all 15 strains, (11 American strains, two Dutch strains, one British and one Japanese isolate,) (Accession No. AF297712 for a Dutch isolate 10783; AF297173 for an American isolate 96–178), together with the published sequence (X68645), were 100% identical [6].

Recently, our research group determined the 16S rDNA sequences of three additional strains, a British type strain NCTC11184^T^, an American prototype strain Kentucky 188 and a Japanese strain EQ59 of *T. equigentalis*, and demonstrated sequence differences in the six 16S rDNA sequences including three reference sequences, whose accession numbers are shown in Table 1 [7]. Consequently, at present, six accessible sequences of 16S rDNA from *T. equigenitalis*, whose sequence differences occur at a few nucleotide positions are available.

Therefore, it is important to determine these sequences from additional strains in other countries, if possible, in order to clarify any anomalies regarding 16S rDNA sequence heterogeneity. Therefore, the authors wished to clone and sequence the approximately full-length 16S rDNA from more strains of *T. equigenitalis *isolated in Japan, Australia and France and compare these sequences among the existing published sequences described above.

## Results

As shown in Table 2 [see Additional file 2], the nucleotide sequence differences were demonstrated at six loci in the 1469 nucleotide sequence of the nearly full-length 16S rDNA, among 17 strains (EQ70 ~ Fr-10) isolated in Japan, Australia and France (AB200397–AB200413 in Table 1). Moreover, 12 polymorphic sites occurred among 23 sequences of the 16S rDNA, including the six reference sequences, as demonstrated in Table 2. In relation to the three countries, Japan, Australia and France, the strains from each respective country, interestingly, gave an identical sequence. Moreover, these strains whose 16S rDNA sequences were analyzed, are from all over the world and carried distinct multiple genotypes [3]. In addition, the sequences of two American strains (Kentucky 188 and 96–178 [AF 297173]) used as references in the present study are different on some positions.

Consequently, in the present study, a high sequence similarity of 16S rDNA (99.5% or more) with the nine polymorphic sites was observed, between np 138 and 501, of a total of 12, where substitutions and deletions were noted. Thus, there is a single polymorphic site in the approximately 2/3 of the 16S rDNA sequence between np 502 and 1469, while the first approximately 1/3 between np 1 and 501 has nine polymorphic sites among 17 strains, suggesting an extremely high sequence similarity in the approximately 2/3 downstream of the 16S rDNA sequence of *T. equigenitalis*.

## Discussion

Recently, Jang *et al*. demonstrated that the 16S rDNA of *T. equigenitalis *gave 97.6% sequence similarity to those of *T. asinigenitalis *[6]. In relation to one of the present molecular guidelines, it is suggested that 3% sequence variation of the 16S rDNA sequence is a threshold value to represent distinctly different bacterial species [8-11] However, most recently, some examples of lower levels of 16S rDNA sequence variations were found among some *Campylobacter *species [12]. The organisms within the genus *Taylorella *are also an example of lower levels of 16S rDNA sequence variation.

## Conclusion

The 16S rDNA sequences of *T. equigenitalis *were demonstrated to give high sequence similarity (99.5% or more) to each other among 23 sequences, but were not identical. Lower levels of 16S rDNA sequence variations were confirmed among the organisms of the genus *Taylorella*.

## Methods

### *T. equigenitalis *strains

Seventeen strains of *T. equigenitalis*, which had been isolated in Japan (n = 6), Australia (n = 7) and France (n = 4) and examined in the present study are shown in Table 1 [see Additional File 1]. Although six Japanese strains, seven Australian strains and a French strains (Fr-10) were isolated from mares clinically affected with CEM, sources and symptom of the two French strains (Fr-1 and Fr-2) are not available. Since male horses were demonstrated to transmit the organism responsible for CEM, in the present study an isolate isolated from a symptomless carrier male French Trotter (Fr-9) was also examined (Table 1) [see Additional file 1].

### Cultural conditions and genomic DNA preparation

Culture conditions for these strains and genomic DNA preparation have been described previously [13,14].

### PCR amplification, cloning and sequencing

PCR amplification, cloning and sequencing of the nearly full-length 16S rDNA have also been described [15]. In relation to the PCR, at present, a primer pair set (fD1; 5'-GAATTTGATCCTGGCTCAG-3' and rTel; 5'-GGCTACCTTGTTACGACTT-3') was employed for amplification of the nearly full-length 16S rDNA from *T. equigenitalis *strains. The primer fD1 contained the 3' end-19 nucleotide sequences of the original fD1 primer sequence [16]. The rTel was constructed based on the nucleotide sequence information of the 3' end region of *E. coli* 16S rDNA of *rrnB* operon (DDBJ/EMBL/GenBank Accession No. J01695).

### Nucleotide sequence analysis

The sequences of the nearly full-length 16S rDNA were aligned using GENETYX-MAC version 9.0 (GENETYX Co. Tokyo, Japan). The sequence data of the 16S rDNA of *T. equigenitalis *determined in the present study are accessible in the DDBJ/EMBL/GenBank, as shown in Table 1, as well as the six reference sequences.

The nucleotide sequences corresponding to the primers fD1 and rTel, employed for PCR amplification of the nearly full-length 16S rDNA from the 17 strains of *T. equigenitalis*, were excluded for similarity analysis. Therefore, the nucleotide position (np) one (A) of the sequence data shown in the DDBJ/EMBL/GenBank corresponds to np 28 of the sequence of the 16S rRNA gene from the *rrnB *cistron of *Escherichia coli *[17,18]. As already described [7], in the three reference sequences from NCTC11184^T^, Kentucky 188 and EQ59, CCTCCT sequences (np 1513 to 1518), which are highly conserved sequences close to the 3' end of the mature 16S rRNA and complementary to the Shine-Dalgarno sequence on mRNA [19-21], [22] were demonstrated.

## List of abbreviations used

Abbreviation: CEM, contagious equine equigenitalis metritis, *T. equigenitalis*, *Taylorella equigenitalis*; rDNA, ribosomal DNA

## Authors' contributions

MM participated in design of the study, collected strains, drafted the manuscript and review of the manuscript. AT, SK and TS were involved with cloning, sequencing and analysis of the 16S rDNA sequences from *T. equigenitalis *strains. OM, JEM and BCM participated in its design and coordination, and review of the manuscript.

## Supplementary Material

Additional File 2 Table 1 Click here for file

Additional File 1 Table 2 Click here for file
